# The potential of mecciRNA in hepatic stellate cell to regulate progression of nonalcoholic hepatitis

**DOI:** 10.1186/s12967-022-03595-1

**Published:** 2022-09-04

**Authors:** Boqiang Liu, Yuanshi Tian, Jing He, Qiuxia Gu, Binghan Jin, Hao Shen, Weiqi Li, Liang Shi, Hong Yu, Ge Shan, Xiujun Cai

**Affiliations:** 1 Department of General Surgery, Sir Run Run Shaw Hospital, School of Medicine, Zhejiang University, Hangzhou, 310016 China; 2 Zhejiang Provincial Key Laboratory of Laparoscopic Technology, Zhejiang University, Hangzhou, 310016 China; 3 Zhejiang Minimal Invasive Diagnosis and Treatment Technology Research Center of Severe Hepatobiliary Disease, Hangzhou, 310016 China; 4 Zhejiang Research and Development Engineering Laboratory of Minimally Invasive Technology and Equipment, Hangzhou, 310016 China; 5 Zhejiang University Cancer Center, Zhejiang University, Hangzhou, 310030 China; 6 Department of Diagnostic Ultrasound & Echocardiography, Sir Run Run Shaw Hospital, School of Medicine, Zhejiang University, Hangzhou, 310016 China; 7 Department of Endocrinology, The Children’s Hospital, School of Medicine, National Clinical Research Center for Child Health, Zhejiang University, Hangzhou, 310053 China; 8 Department of Pulmonary and Critical Care Medicine, Regional Medical Center for National Institute of Respiratory Diseases, Sir Run Run Shaw Hospital, School of Medicine, Zhejiang University, Hangzhou, 310016 China; 9 Department of Clinical Laboratory, First Affiliated Hospital of the USTC, Chinese Academy of Sciences (CAS) Key Laboratory of Innate Immunity and Chronic Disease, School of Basic Medical Sciences, Division of Life Science and Medicine, University of Science and Technology of China (UTSC), Hefei, 230027 China

**Keywords:** NASH, HSC, circRNA, mecciRNA, miRNA, Immunotyping

## Abstract

**Background:**

Nonalcoholic steatohepatitis (NASH) occupies a substantial proportion of chronic liver disease worldwide, of which pathogenesis needs further research. Recent studies have demonstrated the significant roles of circular RNAs (circRNAs) in NASH, while the function of a novel type of circRNAs, namely mitochondria-encoded circRNAs (mecciRNAs), remains elusive. Therefore, we aimed to investigate their potential to regulate the progression of NASH in this study.

**Methods:**

GSE134146 was used to screen for differentially expressed mecciRNAs in NASH, while GSE46300 was used to identify NASH-related genes. To establish the mecciRNA-miRNA-mRNA networks, circMINE and miRNet databases were used for predicting downstream targets. Then, consensus clustering analysis was used to determine immune subtypes of NASH. Finally, we successfully validated our findings in vitro (LPS-treated hepatic stellate cells [HSCs]) and in vivo (MCD-diet mice) NASH models.

**Results:**

We confirmed that circRNomics balance is disrupted in HSCs of NASH, while two mecciRNAs (hsa_circ_0089761 and hsa_circ_0089763) could function as competing for endogenous RNAs (ceRNAs) to regulate fibrosis-related signals. Furthermore, we constructed two ceRNA networks based on mecciRNAs for the first time. Cell and animal NASH models validated our findings that c-MYC and SMAD2/3 were upregulated in HSCs, while THBS1 and p-STAT3 were upregulated in hepatocytes. Moreover, we identified 21 core genes by overlapping the differentially expressed genes (NASH vs. Normal) with mecciRNA-targeted genes. According to their expression profiles, NASH patients could be divided in 2 different clusters, in which proinflammatory signals (TNF and IL-17 pathways) are significantly activated in Cluster 1.

**Conclusion:**

We successfully established two novel mecciRNA-miRNA-mRNA networks in HSCs and hepatocytes, which were further confirmed by in vitro and in vivo models. Meanwhile, the novel immunotyping model revealed the heterogeneity of NASH, thereby might guiding treatment options. Altogether, our study brought a distinct perspective on the relationship between mecciRNAs and NASH.

**Supplementary Information:**

The online version contains supplementary material available at 10.1186/s12967-022-03595-1.

## Background

Nonalcoholic steatohepatitis (NASH) is the inflammatory subtype of nonalcoholic fatty liver disease (NAFLD), mainly caused by excess lipid accumulation in the liver [1]. According to statistical data, about 30% of the patients with fatty liver would progress to NASH [2]. Histologically, NASH is the manifestation of a wound-healing response to hepatocyte lipotoxicity [3]. Hence, NASH patients might benefit from treatment with preventing lipotoxicity or attenuating repair response effects. Currently, there are several innovative drugs for NASH. For instance, OCA, elafibranor, selonsertib, and CVC have entered phase III trials, despite the controversy about their long-term safety and effects [4–7].

The past two decades have witnessed dramatic advances in understanding the pathogenesis of NASH, in which hepatic stellate cells (HSCs) were identified as the major fibrogenic cells [8]. In the inactive state, HSCs maintain a non-proliferative, quiescent phenotype. However, HSCs become activated upon liver injury, transdifferentiating from vitamin-A-storing cells to myofibroblasts, which are proliferative, contractile, inflammatory, and chemotactic, while also characterized by enhanced ECM production [9]. In addition to this, the role of crosstalk between HSCs and hepatocytes also cannot be ignored, such as the activation of HSCs in response to apoptotic hepatocytes [10]. In summary, further studies exploring the cellular and molecular mechanisms of NASH will help to develop new treatment strategies.

Circular RNAs (circRNAs) are a class of non-coding RNAs that play important roles in several liver diseases, including NASH [11–14]. Among them, mitochondria-encoded circRNAs (mecciRNAs) are a novel type of circRNAs identified recently [15]. Our group demonstrated that mecciRNAs are distributed both inside and outside the mitochondria, despite the mechanism that how they shuttle in and out of mitochondria remains unclear [15, 16]. A striking mecciRNA, namely circSCAR, has been reported that could alleviate NASH via reducing mROS output [17]. This meaningful work strongly suggested the functional roles of mecciRNAs in NASH.

Here, combining bioinformatics analysis with experimental validation, we provided several lines of evidence that revealed the potential of mecciRNAs in HSCs to regulate NASH progression. Hopefully, this study could broaden our knowledge of circRNAs’ function, especially for mecciRNAs, and contribute to the development of treatment strategies for NASH.

## Materials and methods

### Materials

MCD (methionine/choline deficient) feed and standard feed were purchased from Nantong Trophy Feed Technology Company, MCD feed contains (per 1000 g): amino acid premix (methionine free) 175.7 g, methionine 0 g, choline chloride 0 g, sucrose 431.9 g, dextrin 50 g, corn starch 150.0 g, corn oil 100.0 g, cellulose 30.0 g, mineral mix 52.4 g. All the primers used in this study were synthesized by Tsingke Company and CWBIOTECH, their sequences were shown in Supplementary Materials. All of antibodies used in western blot, including c-MYC (ab32072), SMAD2 (ab40855), SMAD3 (ab40854), THBS1 (ab267388), STAT3 (ab68153), p-STAT3 (Y705, ab267373) and GAPDH (ab8245), were purchased from Abcam.

### Animal samples

8 weeks male C57BL/6 mice were kept in a controlled environment (24 ± 2 °C, 12/12 h day/dark cycle). And mice were randomly divided into 2 subgroups (n = 6): Group 1 was fed with standard diet for 6 weeks (control group); Group 2 was fed with MCD diet for 6 weeks (MCD group). After the treatment mentioned above, mice were fasted for 12 h before being sacrificed. The liver was excised and perfused with saline. One portion of the liver from each mouse was fixed in 4% paraformaldehyde solution for histological analysis. Another portion of the liver was used to prepare liver homogenate for the biochemical analysis.

### Cell lines and culture

Human hepatic stellate cells cell lines LX-2 were gifted by Dr. Xinping Huang, Guangzhou Institute of Biomedicine and Health, Chinese Academy of Sciences (purchased from the Advanced Research Center of Central South University). LX-2 was cultured in Dulbecco's Modified Eagle’s Media (Thermo Fisher Scientific, USA) supplemented with 10% fetal bovine serum (FBS, Thermo Fisher Scientific, USA) in a 5% CO2 humidified incubator at 37 °C. All the experiments were performed within 1 months of resuscitation and the cell passage was less than 3 generations from initial resuscitation (avoid activation of LX-2 cells).

### RNA extraction and quantitative real-time polymerase chain reaction (RT-qPCR)

Total RNAs were isolated using Trizol reagent (Invitrogen, USA), either from cultured cells or liver tissue. 2 ug of total RNA was subjected to reverse transcription using Superscript III transcriptase (Invitrogen, USA). RT-qPCR was conducted using a Bio-Rad CFX96 system (Bio-Rad, USA) with SYBR green to determine the expression level of targets of interest. In addition, miRNA cDNA Synthesis Kit (CWBIOTECH, CN) and miRNA qPCR Assay Kit (CWBIOTECH, CN) were used for miRNA detection. Expression levels of circRNAs were normalized to the expression levels of GAPDH, while small RNA RNU6 (U6) was used for miRNA. And GAPDH and MTCO2 served as the cytosolic and mitochondrial control, respectively.

### Mitochondria isolation

We conducted this experiment according to the kit protocol (Cat. no. 37612, QIAGEN, GER). First, LX-2 cells were suspended in Lysis Buffer (selectively disrupts the plasma membrane without solubilizing it, resulting in the isolation of cytosolic proteins), and incubated for 10 min. After that, plasma membranes and compartmentalized organelles, such as nuclei, mitochondria, and endoplasmic reticulum, remained intact and were pelleted by centrifugation (1000×*g*, 10 min). Then, the pellet was resuspended in Disruption Buffer, repeatedly passed through a narrow-gauge needle, and recentrifuged (1000×*g*, 10 min) to pellet nuclei, cell debris, and unbroken cells. The supernatant (contains mitochondria) was recentrifuged (6000×*g*, 10 min) to pellet mitochondria. After removal of the supernatant, mitochondria are washed and resuspended in Mitochondria Storage Buffer.

### Screening of differentially expressed circRNAs and genes

We downloaded high-throughput sequencing and noncoding RNA expression profiling data (GSE134146 and GSE46300) from the GEO database. Among them, GSE134146 was used to screen for differentially expressed mecciRNAs in NASH, while GSE46300 was used to identify NASH-related genes. Differential expressions of all genes were calculated using the R package “limma”, and significance was evaluated by one-way analysis of variance (ANOVA).

### Prediction of circRNAs and miRNAs downstream targets

We used the circMINE database (http://hpcc.siat.ac.cn/circmine/), which is based on 3 well-annotated databases, including miRanda [18], miRBase [19], and circBase resources [20], to predict the potential miRNAs targeted by mecciRNAs.

In addition, miRNet database (https://www.mirnet.ca/) was based on the 14 open-source databases, which could analyze and generate miRNA-mRNA network online. Specifically, the miRNA target gene data were collected from three well-annotated database miRTarBase [21], TarBase [22], and miRecords [23]. The miRNA to molecule interaction data were collected from SM2miR [24] and Pharmaco-miR [25]. The miRNA to disease interaction data were collected from miR2Disease [26] and PhenomiR [27]. The miRNA to epigenetic modifier interaction data were collected from EpimiR [28]. And, the exosomal miRNA annotation data were collected from ExoCarta [29]. It should be emphasized that Protein–Protein Interaction (PPI) database was included in the analysis during the construction of miRNA-mRNA network.

### GO enrichment and KEGG pathway enrichment analysis

GO terms in three categories (GO: biological process, GO: cellular component and GO: molecular function) were used for pathway-enrichment analysis and biological interpretation. We used the GO enrichment analysis and visualization tool (GOrilla) to identify GO terms that were significantly enriched in the target gene list. KEGG pathway enrichment analysis was performed with clusterProfiler with a background set of all entrez IDs mapped to a KEGG pathway.

### Construction of protein–protein interaction network

Given that searching for protein interactions and interaction networks is integral to further exploration of cellular states, biological processes and functions, relevant targets were entered into STRING (version 11.5, https://string-db.org/) [30]. Searching by gene name, selecting Homo sapiens as the species and selecting suitable confidence, the purpose of in-depth study of protein–protein interactions can be achieved. Network nodes and edges represent proteins and protein–protein associations, respectively.

### Classification of NASH immune subtypes

On the basis of the expression profile, 21 mecciRNA-related genes were clustered by the R packages. Consensus Cluster Plus with reps = 100, p Item = 0.8, and p Feature = 1 [31]. By comprehensively analyzing the consistency matrix and the consistency cumulative distribution function, the optimal partition is defined.

### Calculation of immune infiltration score

To differentiate immune levels between 2 clusters, we used single-sample gene set enrichment analysis (ssGSEA) to characterize 23 types of immune cells in the liver tissues, based on the specific gene signatures of immune cells [32].

### Statistical analysis

The applicable statistical methods were used depending on the type of data. The student’s t-test was used for comparisons between groups. ANOVA for multiple comparisons was used to detect differences amongst the various treatments. All data from three separate experiments at least are presented as mean ± SD. Differences were considered significant for P-values less than 0.05. *P < 0.05, **P < 0.01, and ***P < 0.001.

## Results

### circRNomics balance is disrupted in HSCs of NASH

circRNAs have become a novel research hotspot in RNA biology. They localize in specific subcellular compartments, and play different biological roles [33]. However, the functions of circRNAs in NASH is still elusive. As activation of HSCs is well regarded as the central driver of fibrosis in NASH, we decided to explore the differentially expressed circRNAs in HSCs of NASH patients [8].

**Fig. 1:**
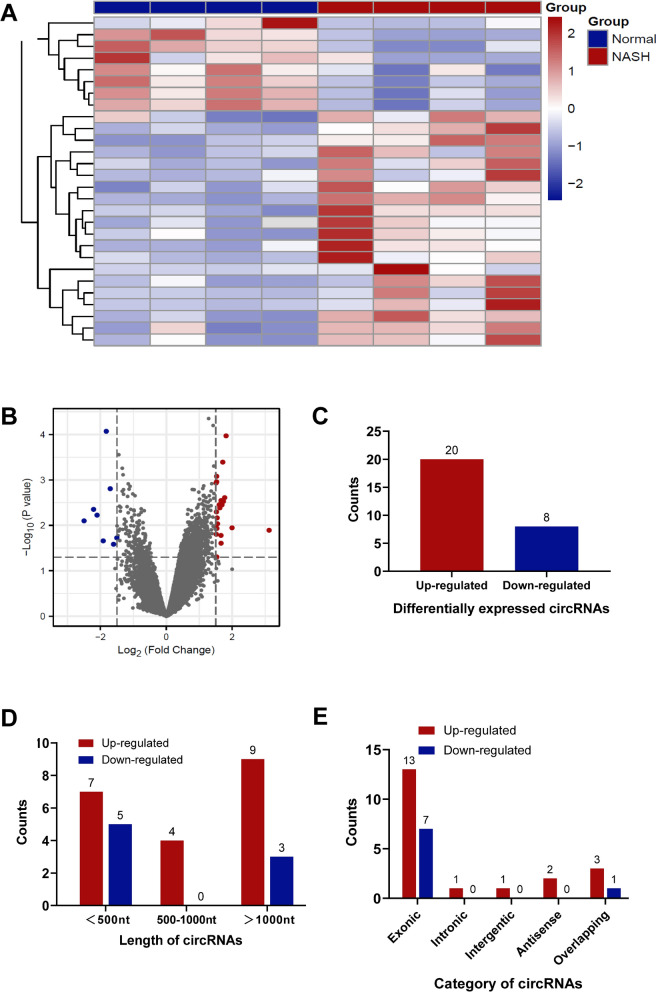
circRNomics Balance is Disrupted in HSCs of NASH. **A, B** The heatmap (**A**) and the volcano plot (**B**) show differentially expressed circRNAs in HSCs of NASH patients (GSE134146). P-value < 0.05 and |Log_2_FC|≥ 1.5. **C** The number of upregulated and downregulated circRNAs, respectively. **D** The length of upregulated and downregulated circRNAs, respectively. **E** The number of upregulated and downregulated circRNAs according to their parental genes

Thus, we downloaded the microarray gene profiling dataset (GSE134146, including 4 patients with NASH cirrhosis and 4 patients without NASH) from Gene Expression Omnibus (GEO) for bioinformatics analysis [17]. The circRNAs with P-value < 0.05 and |Log_2_FC|≥ 1.5 from t-test were identified as differentially expressed circRNAs (Fig. 1A–B). In total, 20 circRNAs were upregulated, while 8 circRNAs were downregulated in NASH (Fig. 1C). Among them, 12 circRNAs are less than 500nt in length (inclined to function as molecular scaffolds), 4 are between 500 and 1000 nt, and 12 are longer than 1000nt (inclined to function as miRNA sponges) (Fig. 1D). Consistent with previous reports, most of them (20 out of 28) are encoded by exons (Fig. 1E). These results indicated that circRNomics homeostasis of HSCs is disrupted in NASH patients. Consequently, we speculated that there might be an association between circRNA imbalance and NASH progression.

### mecciRNAs account for half of downregulated circRNAs in HSCs of NASH

During the development of NASH, there is a constant dysfunction of mitochondria, including alterations in enzyme activities, protein expression, and signaling networks [34]. Thus, we wondered whether the mecciRNAs expression profile was altered in NASH progression.

**Fig. 2:**
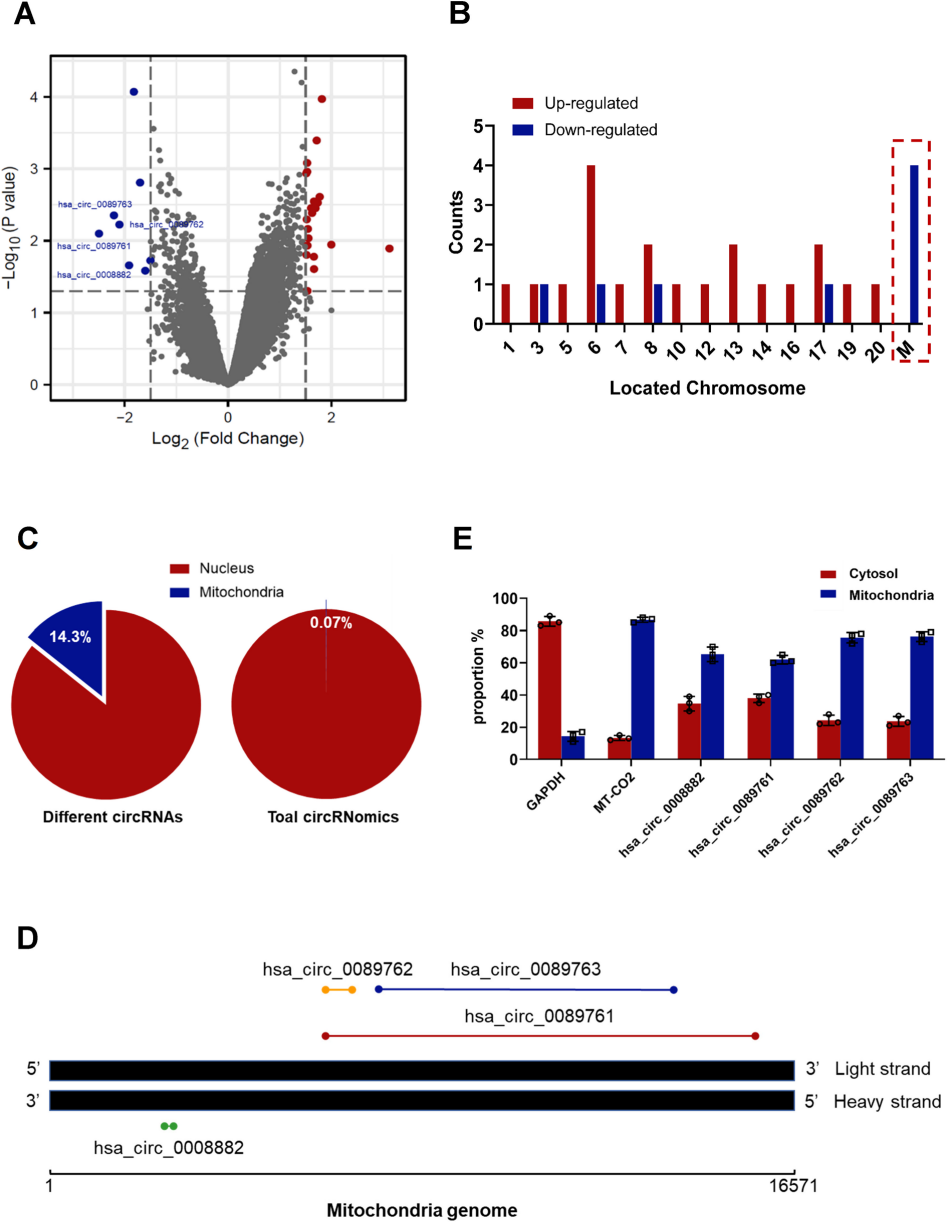
mecciRNAs Account for Half of Downregulated circRNAs in HSCs of NASH. **A** The volcano plot shows upregulated (red) and downregulated (blue) circRNAs in HSCs of NASH patients (GSE134146), in which 4 mecciRNAs were marked. P-value < 0.05 and |Log_2_FC|≥ 1.5. **B** The chromosome locations of differentially expressed circRNAs, in which mecciRNAs were highlighted. **C** The numbers of differentially expressed nucleus-encoded and mitochondria-encoded circRNAs were 24 and 4, respectively (left panel). And the proportion of mecciRNAs in the total circRNomics is shown (right panel). **D** The name, length, location of differentially expressed mecciRNAs in the mitochondrial genome. **E** The expression levels of 4 mecciRNAs in cytosolic and mitochondrial fractions of LX-2 cells were determined by RT-qPCR. GAPDH and MTCO2 mRNA served as the cytosolic and mitochondrial control, respectively

Surprisingly, we found that half (4 out of 8) of downregulated circRNAs were encoded by the mitochondrial genome, namely hsa_circ_0089761, hsa_circ_0089762 (also known as circSCAR [17]), hsa_circ_0089763 and hsa_circ_0008882 (Fig. 2A, B). Given the majority of circRNomics are occupied by nuclear-encoded circRNAs (99.93%), it is noteworthy that mecciRNAs accounted for 14.3% of the differentially expressed circRNAs in NASH (Fig. 2C) [35]. Among them, hsa_circ_0008882 are encoded by the heavy strand of mitochondrial genome, while hsa_circ_0089761, hsa_circ_0089762 and hsa_circ_0089763 are generated from the light strand (Fig. 2D). Additionally, their sizes span a wide range, in which lengths of hsa_circ_0089761 and hsa_circ_0089763 are over 5000 nt, while hsa_circ_0089762 and hsa_circ_0008882 are less than 400 nt (Fig. 2D). Then, we used mitochondrial-cytoplasmic separation assays to determine their cellular localization. The results demonstrated that these mecciRNAs were mainly located in mitochondria, while a part of them were also presented in cytoplasm (Fig. 2E).

### mecciRNAs regulate fibrosis-related signaling pathways in HSCs

circRNAs could function as miRNA decoys, which are defined as miRNA sponges that bind miRNAs and prevent them from suppressing their target mRNAs [36]. Noteworthy, circRNAs are generally with relatively few binding sites for miRNAs, the idea of controlling the stability and quantity of miRNAs by circRNAs and achieving measurable effects should be considered with caution [37, 38]. However, hsa_circ_0089761 and hsa_circ_0089763 are highly possible to possess powerful miRNA-sponge capacity, resulted from their long sequence lengths (over 5000 nt). The copy number ratio between specific circRNA and many different targeted miRNAs is relatively small [39]. Hence, considering the great abundance of substrates (miRNAs), hsa_circ_0089761 and hsa_circ_0089763 are not likely to have competitive binding. On the contrary, it is reasonable to speculate that these two circRNAs might have double sponge capacity for specific miRNAs, due to the fact that hsa_circ_0089761's sequence completely contains hsa_circ_0089763's sequence (Fig. 2D).

**Fig. 3:**
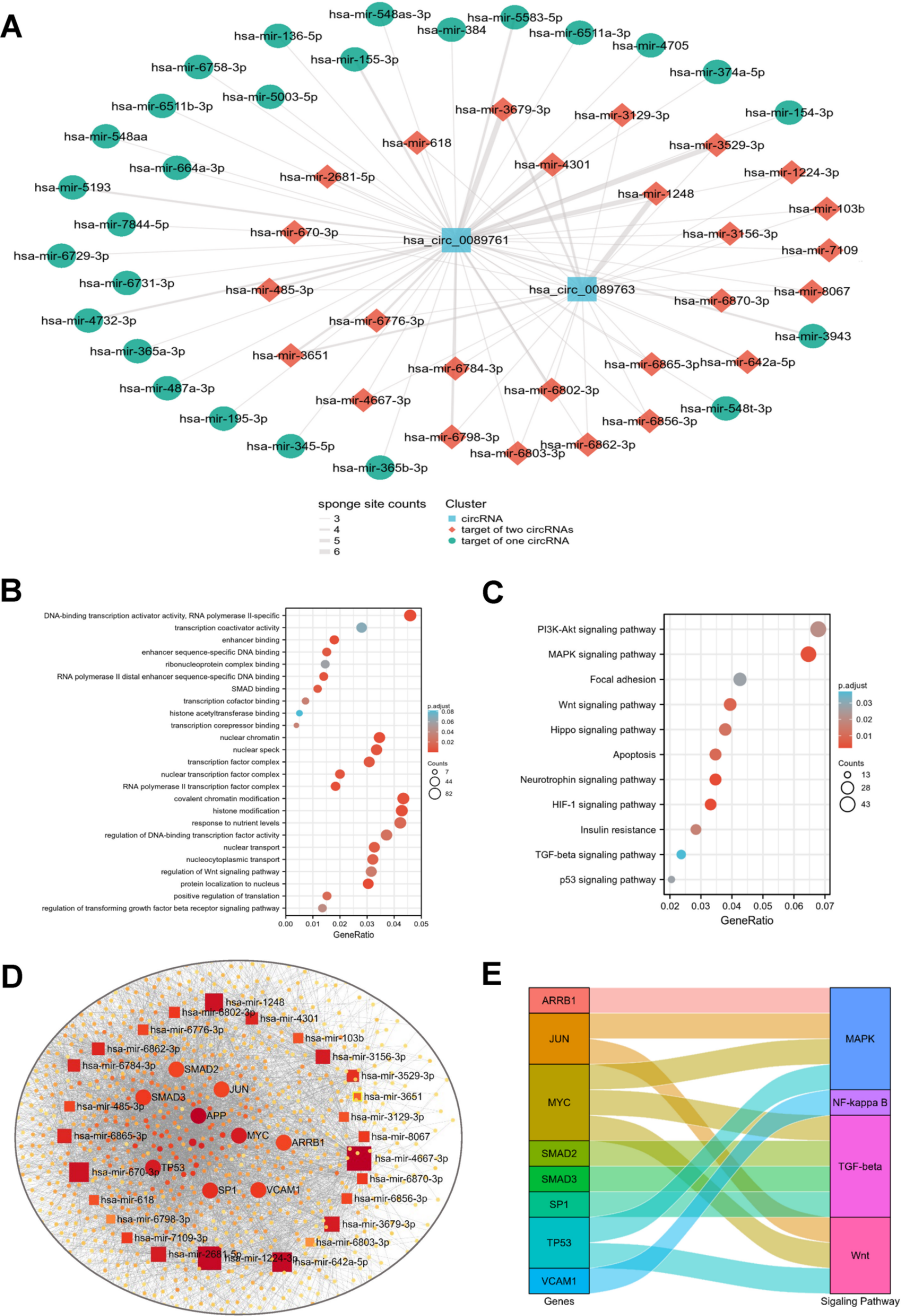
mecciRNAs Regulate Fibrosis-related Signaling Pathways in HSCs. **A** mecciRNA-centered ceRNA network is shown. The thickness of the line represents the number of sponge sites. Color represents the different clusters of ncRNAs. **B, C** The results of GO (**B**) and KEGG (**C**) enrichment analysis for mecciRNAs-related genes were shown, respectively. **D** ceRNA network shows that a set of fibrotic genes (such as SMAD2, SMAD3, MYC, etc.) received the highest enrichment scores. **E** The Sankey diagram shows the relationships between core genes of ceRNA network and NASH-related pathways

First, utilizing the circMINE database, we predicted the miRNAs targeted by hsa_circ_0089761 and hsa_circ_0089763 [40]. According to the filter criteria (a. Score cutoff > 150; b. Energy cutoff < –7; c. Amount of specific miRNA-binding sites ≥ 3), we identified 52 and 26 mecciRNA-miRNA pairs respectively (Fig. 3A). As expected, hsa_circ_0089761 and hsa_circ_0089763 shared 26 identical miRNA targets (hsa-mir-136-5p, hsa-mir-154-3p, hsa-mir-155-3p, hsa-mir-195-3p, hsa-mir-345-5p, hsa-mir-365a-3p, hsa-mir-365b-3p, hsa-mir-374a-5p, hsa-mir-384, hsa-mir-3943, hsa-mir-4705, hsa-mir-4732-3p, hsa-mir-5003-5p, hsa-mir-5193, hsa-mir-548aa, hsa-mir-548as-3p,hsa-mir-548t-3p, hsa-mir-5583-5p, hsa-mir-6511a-3p, hsa-mir-6511b-3p, hsa-mir-664a-3p, hsa-mir-6729-3p, hsa-mir-6731-3p, hsa-mir-6758-3p, hsa-mir-7844-5p, hsa-mir-487a-3p). Then, we identified 2000 mRNAs targeted by the 26 miRNAs in miRNet database (Additional file 4: Table S1) [41]. It is worth mentioning that we also took the PPI database into account when identifying targeted mRNAs. Therefore, among these targets, there may not only be changes in mRNA or protein expression levels, but also changes in protein modification status. Next, To further enhance the reliability, we only selected the part with degree frequency > 1 for subsequent analysis (Additional file 5: Table S2, Additional file 1: Figure S1A). Finally, we successfully established a novel mecciRNA-miRNA-mRNA network in HSCs, including 2 mecciRNA nodes, 26 miRNA nodes and 1343 mRNA nodes (Additional file 2: Figure S1B).

To achieve a better understanding of the functions associated with these mecciRNAs-related genes, we performed GO Term and KEGG pathway enrichment analysis. GO analysis showed an enrichment of GO terms indicative of transcription regulation, such as transcription coactivator activity, enhancer binding, and histone modification (Fig. 3B). Meanwhile, KEGG analysis results revealed the enrichment of several pro-fibrotic signaling pathways, such as Hippo signaling pathway and TGF-β signaling pathway (Fig. 3C) [10, 42]. Strikingly, the top 20 most enriched genes contained many famous NASH-related genes, such as c-MYC, SMAD2 and SMAD3 (Fig. 3D–E) [43–46]. Taken together, these results indicated that mecciRNAs might have the potential to regulate fibrosis-related signaling pathways in HSCs.

### Exosomes-mediated crosstalk between HSCs and hepatocytes in NASH

Death of hepatocytes could induce liver inflammation and then directly or indirectly promote the activation of HSCs [47]. Conversely, activated HSCs also further aggravated the damage and death of hepatocytes [48]. Hence, the crosstalk between HSCs and hepatocytes performs important functions in NASH progression, in which exosome-mediated intercellular communication cannot be ignored [49]. The exosomal cargo consists of proteins, lipids and ncRNAs, while many of the biologic effects of exosomes have been attributed to miRNAs [50]. Therefore, we were particularly interested in whether the changes in mecciRNA-miRNA profile of HSCs could influence the signaling pathways of hepatocytes.

**Fig. 4:**
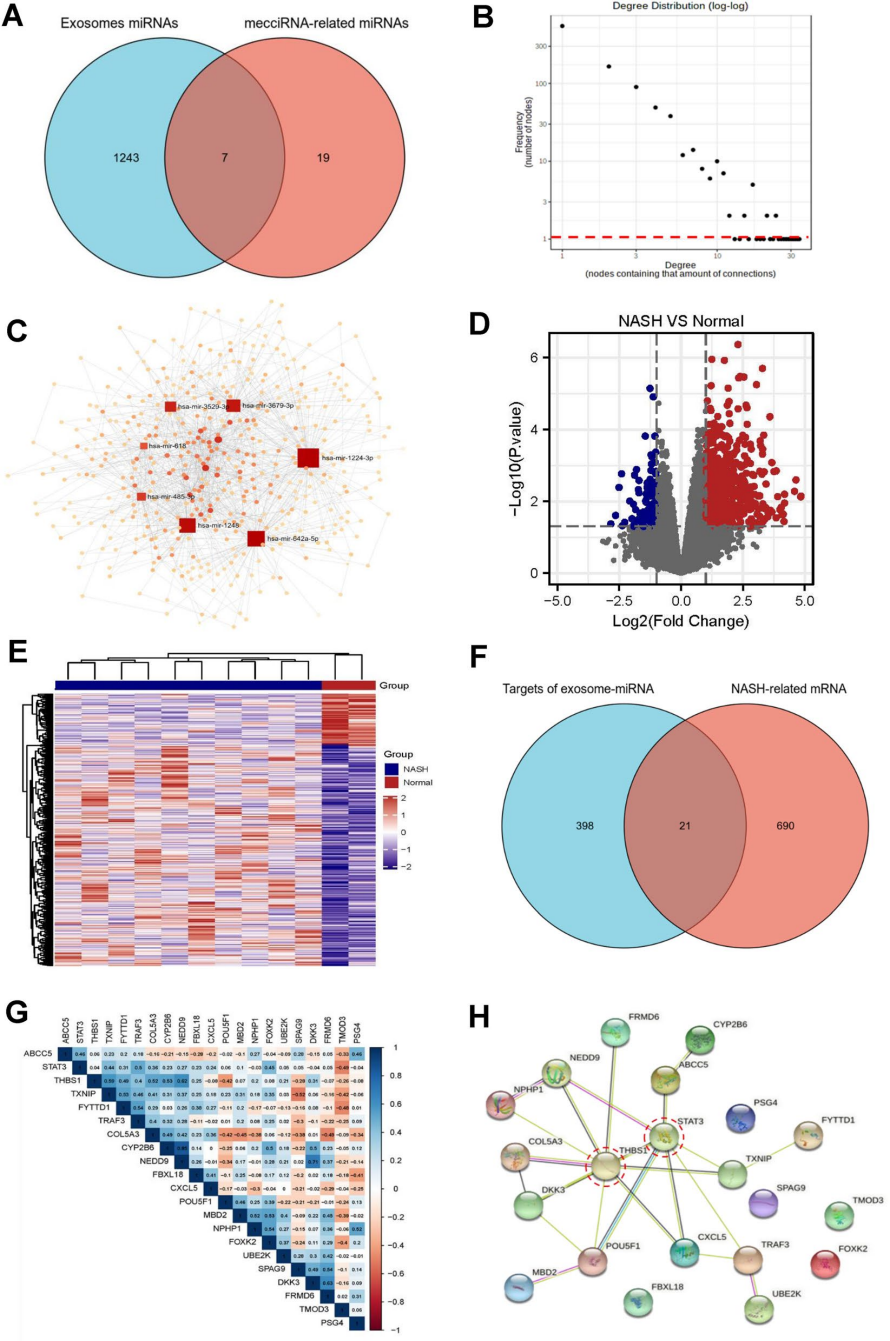
Exosomes-mediated Crosstalk Between HSCs and Hepatic Cells in NASH. **A** The Venn diagram shows that only 7 specific miRNAs possess the potential to regulate NASH-related pathways in hepatocytes. **B, C** State of degree distribution in the ceRNA network (B, frequency [number of nodes] = 1 was highlighted with red dotted line), consisting of 7 miRNAs and 419 mRNAs (C). **D, E** The volcano plot (D) and heatmap (E) shows the differentially expressed genes in NASH liver tissues vs. normal liver tissues (GSE46300, red: upregulated genes, blue: downregulated genes). P-value < 0.05 and |Log_2_FC|≥ 1.0. **F** The Venn diagram shows that there are 21 shared genes between the NASH-related gene set and exosomal-miRNA targeted gene set. **G** Protein interaction network diagram shows the interaction between 21 overlapped proteins. **H** PPI network of 21 overlapped proteins, in which STAT3 and THBS1 (labeled with red circles) are localized at the core of network

First, using the exosomal miRNAs dataset (ExoCarta, a well-known database of exosomal proteins, RNA and lipids) in miRNet, we screened out 7 candidates (hsa-mir-485-3p, hsa-mir-618, hsa-mir-642a-5p, hsa-mir-1224-3p, hsa-mir-1248, hsa-mir-3529-3p and hsa-mir-3679-3p) among the 26 mecciRNA-targeted miRNAs, which had been reported that can be secreted to extracellular microenvironment through exosomes to function (Additional file 6: Table S3, Fig. 4A) [41]. Then, utilizing the same analytical methods and filter criteria, we finally identified 419 mRNAs in hepatocytes, which might be regulated by the mecciRNA-miRNA network (Additional file 7: Table S4, Fig. 4B, C).

Meanwhile, we downloaded the raw sequencing data of GSE46300 from GEO, which includes liver tissues with or without NASH, and obtained a set of differentially expressed genes between NASH and normal liver (|Log_2_FC|≥ 1, P < 0.05, Fig. 4D, E) [51]. In total, 21 overlapped genes were identified (STAT3, POU5F1, TRAF3, UBE2K, CXCL5, SPAG9, MBD2, THBS1, TMOD3, NPHP1, NEDD9, COL5A3, ABCC5, FRMD6, FBXL18, TXNIP, FYTTD1, PSG4, DKK3, CYP2B6, FOXK2), in which STAT3 and THBS1 were localized at the core of protein interaction network (Fig. 4F–H). Despite several reports about their functions in liver steatosis, in-depth molecular mechanisms about STAT3 and THBS1 regulating NASH progression still deserve further study [52–54].

### A novel immunotyping of NASH based on mecciRNA-related network

**Fig. 5:**
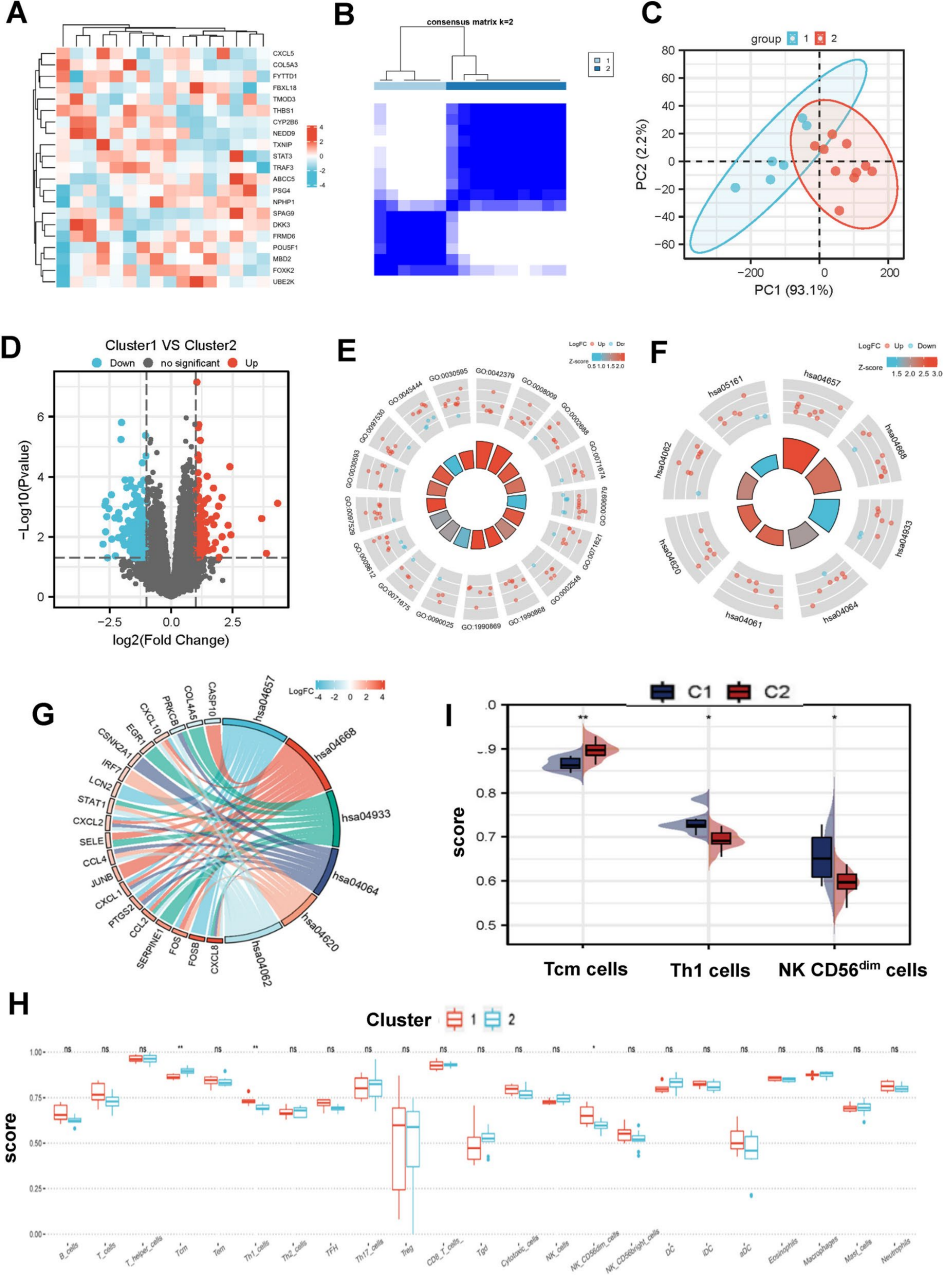
A Novel Immunotyping of NASH Based on mecciRNA-related Network. **A** The heatmap shows the expression profiles of 21 specific targets from 16 steatotic liver tissues. **B** NASH patients clustering heatmap, consensus matrix k = 2. **C** PCA diagram shows the significant differences between 2 clusters of NASH patients (Cluster 1 labeled with blue, Cluster 2 labeled with red). **D** The volcano plot shows the upregulated (red) and downregulated (blue) genes in Cluster 1 *vs.* Cluster 2. P-value < 0.05 and |Log_2_FC|≥ 1.0. **E** The results of GO enrichment analysis for differentially expressed genes between Cluster 1 and Cluster 2. The names of GO terms are shown in Table.1. **F, G** The results of KEGG enrichment analysis for differentially expressed genes between Cluster 1 and Cluster 2. The names of KEGG terms are shown in Table.2. **H, I** Differential enrichment scores of 23 immune cell signatures in Cluster 1 and Cluster 2. *P < 0.05, **P < 0.01, n.s. means none was statistically significant

Classification of specific disease based on different cellular and molecular features could deepen our understanding of this disease and even provide valuable information for treatment. Hence, in order to evaluate the effects of these 21 genes on NASH, we extracted the expression profiles of these targets from GEO46300 to construct a consensus cluster (Fig. 5A). Two subtypes (Cluster 1 and Cluster 2) were obtained with the minimum variance within the group and the maximum variance across the groups (Fig. 5B, Additional file 2: Fig. 2A–D). Meanwhile, the results of principal component analysis (PCA) confirmed that there are significant differences between the two clusters (Fig. 5C).

We then performed differential gene expression analysis across the transcriptomes of these two subtypes. In total, we identified 273 differentially expressed genes (Additional file 8: Table S5, Fig. 5D). GO analysis revealed that these genes were highly enriched for functions related to chemokine activity and immune cell chemotaxis (Table. 1, Fig. 5E). And KEGG analysis demonstrated that, in addition to the upregulation of immune cell chemotaxis-related pathways, IL-17 signaling pathway and TNF signaling pathway were also significantly activated in Cluster 1, which could facilitate the development of NAHS by exerting proinflammatory effects (Table. 2, Fig. 5F–G) [55, 56].

**Table 1:** Results of GO enrichment analysis between Cluster 1 and Cluster 2

Ontology	ID	Description	GeneRatio	BgRatio	P value	p.adjust	Q value
BP	GO:0002688	Regulation of leukocyte chemotaxis	8/224	114/18670	7.01e−05	0.066	0.064
BP	GO:0071674	Mononuclear cell migration	7/224	90/18670	1.05e−04	0.066	0.064
BP	GO:0006979	Response to oxidative stress	16/224	451/18670	1.16e−04	0.066	0.064
BP	GO:0071621	Granulocyte chemotaxis	8/224	123/18670	1.20e−04	0.066	0.064
BP	GO:0002548	Monocyte chemotaxis	6/224	65/18670	1.28e−04	0.066	0.064
BP	GO:1990868	Response to chemokine	7/224	97/18670	1.68e−04	0.066	0.064
BP	GO:1990869	Cellular response to chemokine	7/224	97/18670	1.68e−04	0.066	0.064
BP	GO:0090025	Regulation of monocyte chemotaxis	4/224	25/18670	2.09e−04	0.066	0.064
BP	GO:0071675	Regulation of mononuclear cell migration	5/224	46/18670	2.18e−04	0.066	0.064
BP	GO:0009612	Response to mechanical stimulus	10/224	210/18670	2.32e−04	0.066	0.064
BP	GO:0097529	Myeloid leukocyte migration	10/224	210/18670	2.32e−04	0.066	0.064
BP	GO:0030593	Neutrophil chemotaxis	7/224	104/18670	2.58e−04	0.068	0.065
BP	GO:0097530	Granulocyte migration	8/224	141/18670	3.06e−04	0.074	0.071
BP	GO:0045444	Fat cell differentiation	10/224	223/18670	3.75e−04	0.081	0.078
BP	GO:0030595	Leukocyte chemotaxis	10/224	224/18670	3.88e−04	0.081	0.078
MF	GO:0042379	Chemokine receptor binding	7/218	66/17697	1.64e−05	0.008	0.007
MF	GO:0008009	Chemokine activity	6/218	49/17697	2.93e−05	0.008	0.007

**Table 2:** Results of KEGG enrichment analysis between Cluster 1 and Cluster 2

ID	Description	GeneRatio	BgRatio	P value	p.adjust	Q value
hsa04657	IL-17 signaling pathway	9/97	94/8076	1.64e−06	3.41e−04	3.07e−04
hsa04668	TNF signaling pathway	9/97	112/8076	7.09e−06	7.37e−04	6.64e−04
hsa04933	AGE-RAGE signaling pathway in diabetic complications	8/97	100/8076	2.43e−05	0.002	0.002
hsa04064	NF-kappa B signaling pathway	7/97	104/8076	2.38e−04	0.012	0.011
hsa04620	Toll-like receptor signaling pathway	6/97	104/8076	0.002	0.039	0.035
hsa04062	Chemokine signaling pathway	8/97	192/8076	0.002	0.043	0.038

Considering the differences between 2 subtypes were mainly focused on immune-related pathways, we further utilized ssGSEA scores to characterize the immune cell components in the 2 subtypes [32]. Despite most of the differences did not reach statistical significance, immune cell infiltration was higher in Cluster 1 than in Cluster 2 generally, which was consistent with the GO and KEGG analysis (Fig. 5H). Noteworthy, infiltration of T helper 1 (Th1) cells and NK CD56^dim^ cells was significantly higher in Cluster 1, while central memory T (Tcm) cells infiltration was reduced (Fig. 5I). It is universally agreed that Th1 cells are proinflammatory cells and aggravate liver fibrosis development [57–59]. However, the roles of NK CD56^dim^ cells and Tcm cells in NASH remain elusive and require further study.

In general, we established a novel immunotyping of NASH based on mecciRNA-related network, in which Cluster 1 presents increased immune cell infiltration and activation of proinflammatory signaling pathways.

### Validation of mecciRNAs networks in vitro and in vivo

**Fig. 6:**
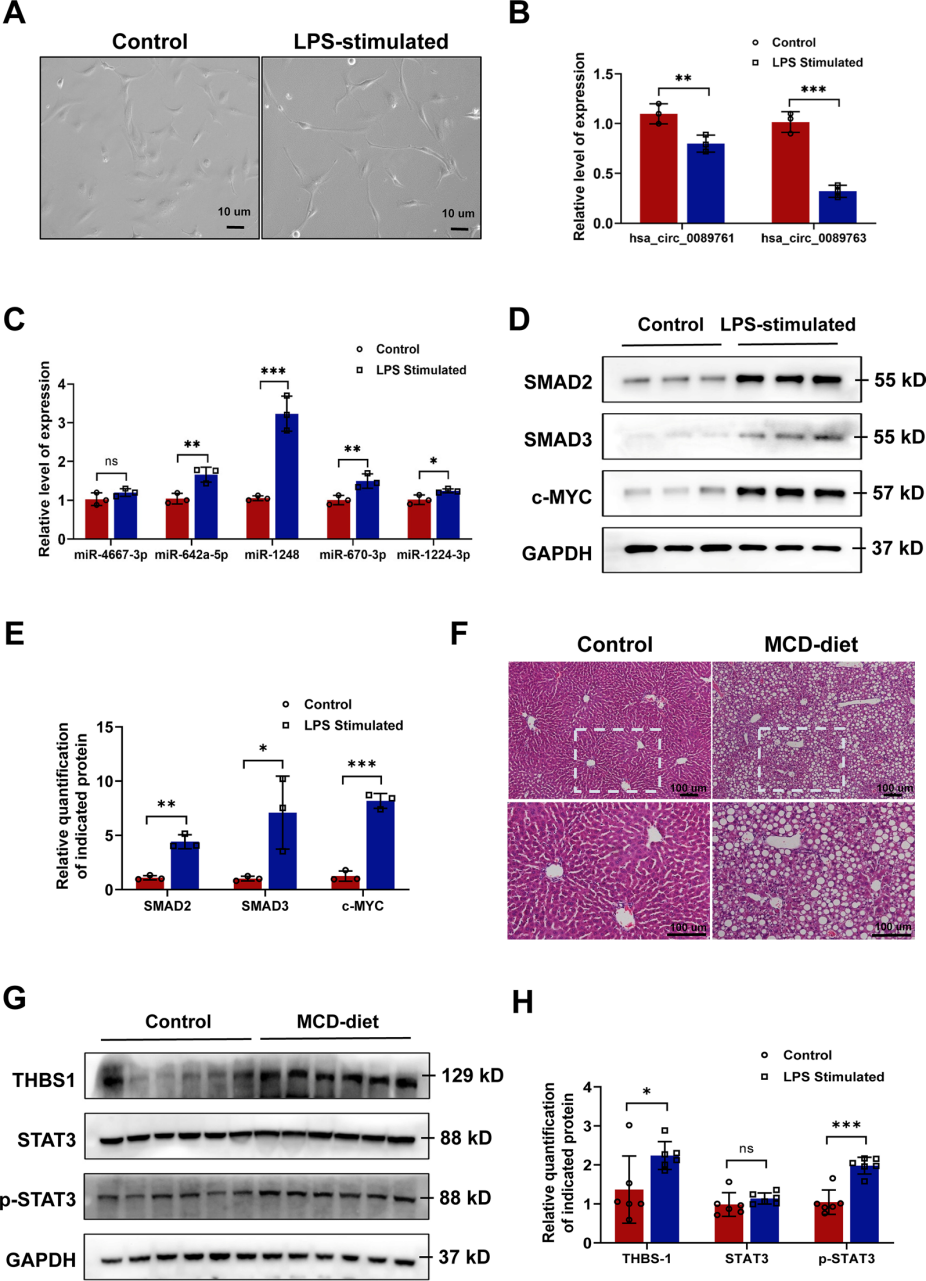
Validation of mecciRNAs Networks in vitro and in vivo. **A** Under the stimulation of LPS (100 ng/ml, 48 h), LX-2 cells were significantly activated, and their cell morphology changed. Scale bar = 10 μm. **B** RT-qPCR result shows that the expression levels of hsa_circ_0087761 and hsa_circ_0087763 were decreased in activated LX-2 cells. The expression of GAPDH was used as the reference in the RT-qPCR analyses. **P < 0.01, ***P < 0.001. **C** RT-qPCR result shows that miR-642a-5p, miR-1248, miR-670-3p, and miR-1224-3p were upregulated significantly in activated LX-2 cells, while the expression level of miR-4667-3p was not changed. The expression of U6 was used as the reference in the RT-qPCR analyses. *P < 0.05, **P < 0.01, ***P < 0.001, n.s. means none was statistically significant. **D, E** Endogenous levels of SMAD2, SMAD3, and c-MYC from control or LPS-stimulated LX-2 cells (n = 3) were determined by Western blot (**D**). GAPDH was used as a loading control. The relative ratios of indicated proteins over GAPDH and statistical analysis results are shown (**E**). *P < 0.05, **P < 0.01, ***P < 0.001. **F** HE stained images shows that the mice in MCD-diet group had significant hepatic steatosis compared to the mice in standard diet group. Scale bar = 100 μm. **G, H** Endogenous levels of THBS1, STAT3, and p-STAT3 from control or MCD-diet mice (n = 6) were determined by Western blot (**G**). GAPDH was used as a loading control. The relative ratios of indicated proteins over GAPDH and statistical analysis results are shown (**H**). *P < 0.05, **P < 0.01, ***P < 0.001, n.s. means none was statistically significant

First, to validate the bioinformatic conclusions, we successfully established HSC-activation model by exposing LX-2 cells (human hepatic stellate cell line) to lipopolysaccharide (LPS) at a concentration of 100 ng/ml for 48 h (Fig. 6A). We confirmed that both hsa_circ_0089761 and hsa_circ_0089763 was detected in a lower amount in activated HSCs compared to wild-type HSCs (Fig. 6B). To further validate our computational predictions, we detected the expression levels of 5 core miRNAs (as test cases) in the mecciRNA-miRNA network. The results largely conformed to our predictions that miR-642a-5p, miR-1248, miR-670-3p and miR-1224-3p was upregulated in activated HSCs, while only miR-4667-3p level was comparable between the two groups (Fig. 6C). As shown in Fig. 3D, SMAD2, SMAD3 and c-MYC are core targets in HSCs mecciRNA-miRNA network, which have been reported to play vital roles in NASH progression [43–46]. The western blot results demonstrated the significant upregulation in expression levels of these proteins in activated HSCs as expected (Fig. 6D).

Meanwhile, a set of candidates were considered as the downstream effectors of mecciRNAs-miRNAs network in hepatocytes, in which THBS1 and STAT3 occupied key positions. As feeding mice an MCD diet is a widely accepted model to study relevant mechanisms in NASH, we established this animal model, and detected the expression levels of THBS1 and STAT3 in control group and MCD-diet group (Fig. 6E) [60, 61]. The results revealed that the expression of THBS1 was increased in MCD-diet group, despite the similar expression levels of STAT3 between the two groups (Fig. 6F). Considering PPI database was also included in mecciRNA network analysis, we further detected the expression level of phosphorylated STAT3. As shown in Fig. 6F, the results demonstrated the elevated level of phosphorylated STAT3 in MCD-diet group.

Based on these experimental results, we confirmed the reliability of mecciRNA-miRNA networks preliminarily. Additionally, our study also brought a distinct perspective on the understanding of relationship between mecciRNAs and NASH.

## Discussion

With the development of society and economy, the incidence of NASH has gradually increased, becoming one of the main chronic diseases globally [62]. Therefore, clarification the cellular and molecular mechanism of NASH has become a research hotspot. Over the course of research, it was found that circRNAs might function significantly in progression of NASH, in which a novel type of circRNAs, namely mecciRNAs, have attracted much interest recently [14, 17, 63]. Through bioinformatic analysis and experimental verification, this study has proposed the possibility that mecciRNAs might function as ceRNAs to regulate NASH progression.

Although our group demonstrated the existence of mecciRNAs and provided solid evidence to confirm that mecciRNAs are also localized outside of mitochondria [15], additional studies are needed to further illuminate several issues.

First, the mechanism by which mecciRNAs shuttle in and out of mitochondria is not well understood. Our previous study revealed that mecciRNAs could interact with PNPase, an enzyme that has been shown to be critical for the mitochondrial import of several noncoding RNAs [15, 64–66]. Hence, we speculated that the mitochondrial export of mecciRNAs might require the participation of PNPase. In addition, members of the mitochondrial carrier family (SLC25) provide the transport steps for nucleotides across the mitochondrial inner membrane, approximately one-third of which are currently orphan transporters, with no known substrate [67]. Hence, it is reasonable to speculate that SLC25 family might mediate mecciRNAs transport.

Second, the biosynthesis and metabolism of mecciRNAs require more in-depth research. mecciRNAs and nuclear-encoded circRNAs possess similar junction motifs, suggesting a mechanism of back splicing might exist in mecciRNA biogenesis. Although previous studies believed that introns and linear splicing events cannot occur in the mitochondria of multicellular animals, recent study has found that splicing factors may exist in mammalian mitochondria [68]. Additionally, our data showed that nuclear-transfected plasmids harboring the corresponding mitochondrial DNA fragments (with the flanking sequences) can successfully overexpress mecciRNA [15]. Then it may be speculated that there is a back splicing form of mitochondrial splicing in multicellular animals to generate mecciRNAs. Meanwhile, the discovery of nuclear-encoded circRNAs generated from single-exon genes by back splicing, and no linear splicing is involved in the biogenesis of mRNA from single-exon genes also make us reasonably suspect that mecciRNAs may be generated through a unique splicing-independent mechanism [20].

Both of hsa_circ_0089761 and hsa_circ_0089763 possess strong ability of miRNA sponges, due to their surprising sequence lengths and partial cytoplasmic localization. And incorporating PPI database into analysis made the bioinformatic prediction much closer to reality, which set this work apart from the other ceRNA network studies. It also meant that the targets of mecciRNA-miRNA networks may not only experience changes in expression levels, but also may undergo changes in protein modification due to the effect of other proteins (such as phosphorylation, etc.). Meanwhile, as the tools matures for ceRNA network analysis, utilizing bioinformatic methods to predict the potential functions of circRNAs is becoming more and more reliable [69]. Building on this theoretical foundation, we carried out this study.

Completely different from the two mecciRNAs mentioned above, hsa_circ_0089762 and hsa_circ_0008882 is really tiny, so that might act as molecular scaffolds to regulate specific complex functions or serve as molecular chaperones in the folding of mitochondria-imported proteins [15, 36]. Further study is certainly required to confirm this speculation. As of now, there is only one mecciRNA has been reported that could perform significant effect on NASH [17]. Considering the major contribution of mitochondria dysfunction in NASH, it is reasonable to speculate that a substantial proportion of functional mecciRNAs remain unidentified [70].

In this study, we have divided NASH patients into 2 subtypes according to 21 mecciRNA-related genes. Unexpectedly, we found large differences in immune-related signaling between the two subtypes. As we all know, NASH is essentially a chronic disease of immunometabolism, whose progression is associated with the liver immune microenvironment [71]. From this perspective, it is of significant interest that a set of mecciRNA-related genes could guide immunophenotyping in NASH, of which molecular mechanism deserves further research. Unfortunately, GSE46300 lacks clinical information of patients, such as liver enzymes. Hence, it is not clear whether the clinical manifestations of cluster 1 patients are more severe than those of cluster 2, in addition to the up-regulation of proinflammatory signals.

However, our study still has some limitations. On the one hand, due to the significant differences existing between human and mice, especially in transcript level, mice model of NASH could not fully simulate the complexity of human illnesses [72, 73]. Interestingly, there are currently models using humanized liver in mice that allow to study NASH development, which might make study more closely with the reality [74]. On the other hand, compared with the next generation sequencing used in this study, single-cell RNA sequencing, as a novel method to comprehensively characterize the cells, could better reflect the changes of hepatocytes and hepatic stellate cells during the progression of NASH, which have a broad application prospect in NASH-related research.

## Conclusion

We successfully established two mecciRNA-miRNA-mRNA networks based on bioinformatic analysis. Moreover, LPS and MCD-diet induced NASH model supported our prediction to some extent. Meanwhile, utilizing 21 NASH-related genes targeted by mecciRNAs, a novel immunotyping model for NASH was built for the first time, directly reflecting the state of liver immune microenvironment, which might guide treatment option in future. In summary, our study brought a distinct perspective on the relationship between mecciRNAs and NASH.

## Supplementary Information

**Additional file 1: Supplementary Figure. 1 mecciRNAs Regulate Fibrosis-related Signaling Pathways in HSCs**

**Additional file 2: Supplementary Figure. 2 A Novel Immunotyping of NASH Based on mecciRNA-related Network**

**Additional file 3:** **Supplementary Figure Legends and Primer Sequence.** 

**Additional file 4: Supplementary Table. 1 The 2000 mRNAs Targeted by 26 miRNAs in miRNet Database.**

**Additional file 5: Supplementary Table. 2 The 1343 mRNAs Targeted by 7 exosomal miRNAs in miRNet Database.**

**Additional file 6:** **Supplementary Table. 3 .** **The** **Evidence** **Indicates** **The** **Presence** **of** **These Following 7 miRNAs in Exosomes.**

**Additional file 7: Supplementary Table. 4  The 419 mRNAs Regulated by mecciRNA-miRNA Network in Hepatocytes.**

**Additional file 8: Supplementary Table. 5 The 273 Differentially Expressed Genes Between Cluster 1 and Cluster 2.**

## Declarations

### Availability of data and materials

The data sets analysed during this study are available in public, open access repositories listed in this article.
