# Supplementary figures and images for: The potential of mecciRNA in hepatic stellate cell to regulate progression of nonalcoholic hepatitis
Source: J Transl Med. 2022 Sep 4;20:393. doi: 10.1186/s12967-022-03595-1 (PMC9441041; doi:10.1186/s12967-022-03595-1)

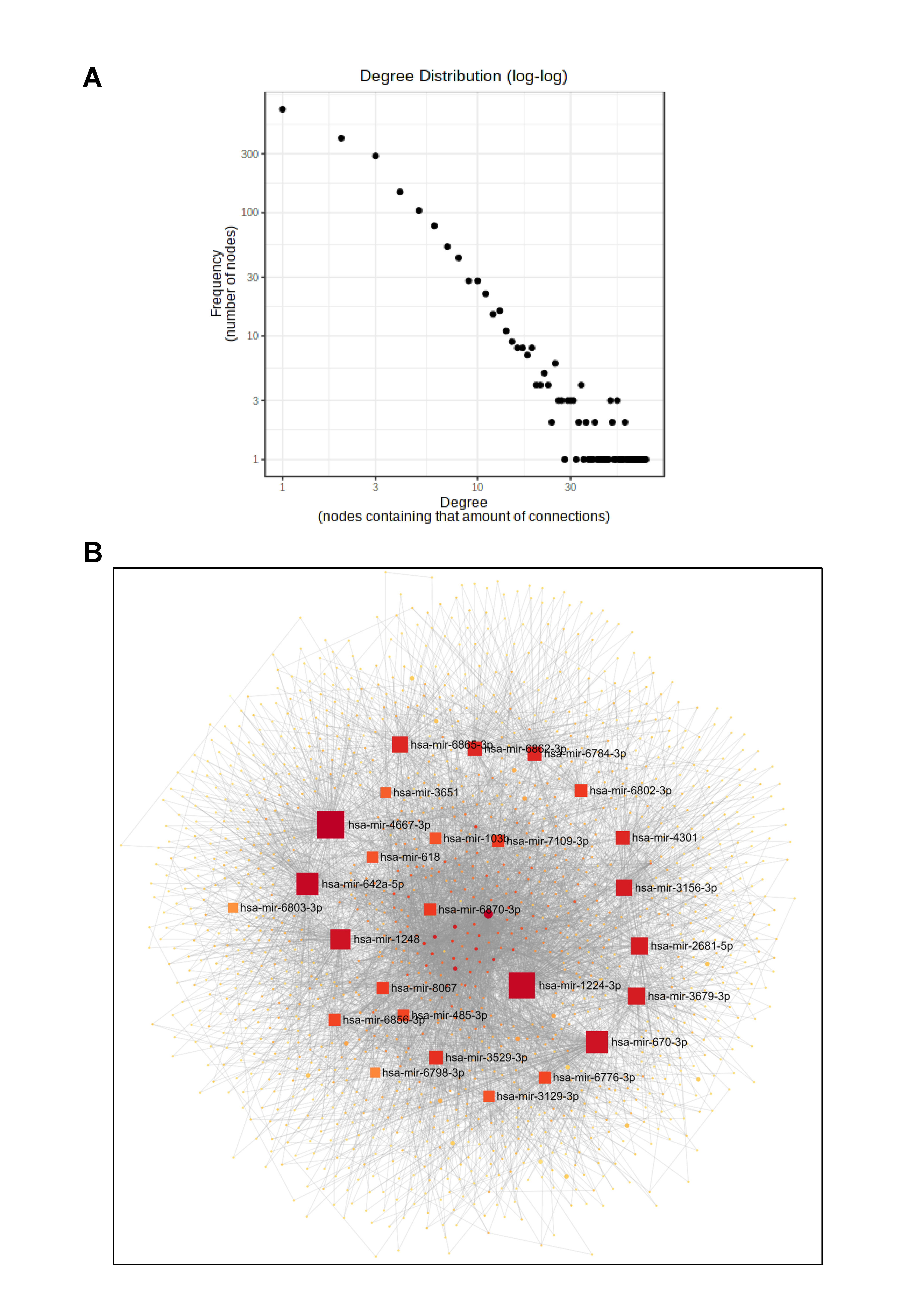

Supplement: Supplementary file 1 — Additional file 1: Supplementary Figure. 1 mecciRNAs Regulate Fibrosis-related Signaling Pathways in HSCs [file 12967_2022_3595_MOESM1_ESM.tif]

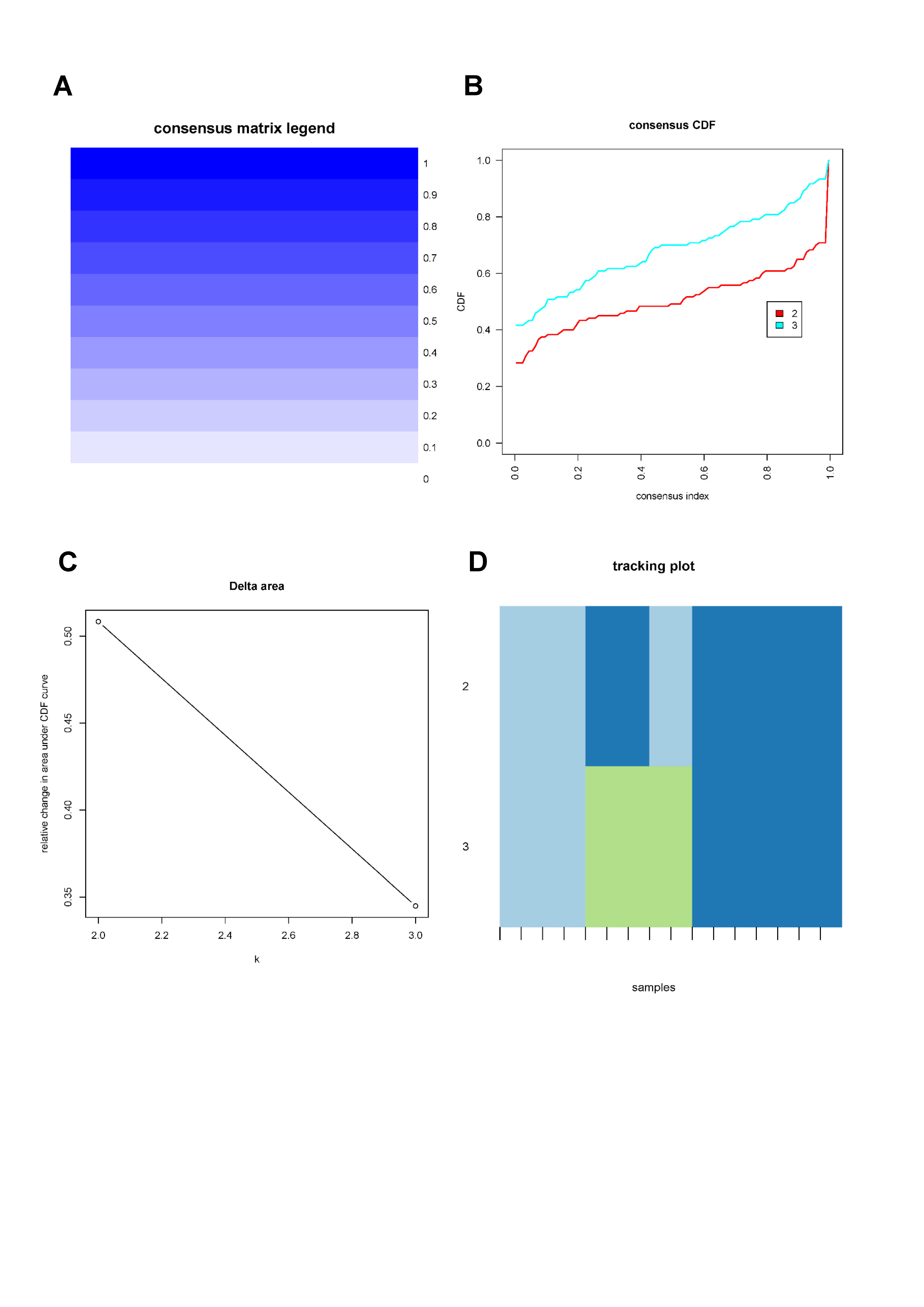

Supplement: Supplementary file 2 — Additional file 2: Supplementary Figure. 2 A Novel Immunotyping of NASH Based on mecciRNA-related Network [file 12967_2022_3595_MOESM2_ESM.tif]
