# Supplementary material for: The potential of mecciRNA in hepatic stellate cell to regulate progression of nonalcoholic hepatitis
Source: J Transl Med. 2022 Sep 4;20:393. doi: 10.1186/s12967-022-03595-1 (PMC9441041; doi:10.1186/s12967-022-03595-1)
Supplement: Supplementary file 3 — Additional file 3: Supplementary Figure Legends and Primer Sequence. [file 12967_2022_3595_MOESM3_ESM.docx]

Additional file

**1. Additional file Figure Legends**

**Figure S1 mecciRNAs Regulate Fibrosis-related Signaling Pathways in HSCs**

**A and B.** State of degree distribution in the ceRNA network of HSCs (A), consisting of 26 miRNAs and 1343 mRNAs (B).

**Figure S2 A Novel Immunotyping of NASH Based on mecciRNA-related Network.**

**A.** Consensus matrix legend of 21 specific genes in NASH cohort.

**B.** Cumulative distribution function curve of 21 specific genes in NASH cohort.

**C.** Delta area of 21 specific genes in NASH cohort.

**D.** Tracking plot of 21 specific genes in NASH cohort.

**2. Primer Sequence**

| **Name** | **Sequence** | **Supplier** |
| --- | --- | --- |
| hsa_circ_0008882-F | AGCATTAGCAGGAATACCTTT | TsingKe |
| hsa_circ_0008882-R | AGGCTGCCAATGGTGAGGGA | TsingKe |
| hsa_circ_0089761-F | GCGCTGCATGTGCCATAGGC | TsingKe |
| hsa_circ_0089761-R | GGAGGACAACCAGTAAGCTACC | TsingKe |
| hsa_circ_0089762-F | GCTGCATGTGCCATGTCGCC | TsingKe |
| hsa_circ_0089762-R | GGCCACCAATGGTACTGAACC | TsingKe |
| hsa_circ_0089763-F | GGTGGTAGTTTGTGTTTAATAT | TsingKe |
| hsa_circ_0089763-R | CTACTCCTAATCACATAAATGC | TsingKe |
| GAPDH-F | ATCACCATCTTCCAGGAGCGA | TsingKe |
| GAPDH-R | CCTTCTCCATGGTGGTGAAGAC | TsingKe |
| MTCO2-F | CTGCGACTCCTTGACGTTGAC | TsingKe |
| MTCO2-R | GTAGCGGTGAAAGTGGTTTGGT | TsingKe |
| hsa-miR-4667-3p-F | TCCCTCCTTCTGTCCCCACAG | TsingKe |
| hsa-miR-642a-5p-F | GTCCCTCTCCAAATGTGTCTTG | TsingKe |
| hsa-miR-1248-F | ACCTTCTTGTATAAGCACTGTGCTAAA | TsingKe |
| hsa-miR-670-3p-F | TTTCCTCATATTCATTCAGGA | TsingKe |
| hsa-miR-1224-3p-F | CCCCACCTCCTCTCTCCTCAG | TsingKe |
| Universal RT-qPCR Reverse Primer | Included with the kit | CWBIOTECH |
| Universal Reverse Transcription Primer | Included with the kit | CWBIOTECH |
